# Trends and Applications in Computationally Driven Drug Repurposing

**DOI:** 10.3390/ijms242216511

**Published:** 2023-11-20

**Authors:** Luca Pinzi, Giulio Rastelli

**Affiliations:** Department of Life Sciences, University of Modena and Reggio Emilia, Via Giuseppe Campi 103, 41125 Modena, Italy; luca.pinzi@unimore.it

Drug repurposing is a widely used approach originally developed to aid in the identification of new uses of already existing drugs outside the scope of the original medical indication [1]. More recently, it has been extended to include pre-clinical and clinical candidates, already synthesized compounds, and natural products [2,3], allowing their full exploitation in drug discovery and development. Drug repurposing presents several advantages with respect to traditional de novo design, including lower costs, significantly shortened pre-clinical timelines and reduced safety concerns, which often hamper the obtainment of valuable clinical candidates [1,2,3]. So far, drug repurposing has mainly originated from retrospective (often serendipitous) analysis of clinical and pharmacological effects derived from unexpected off-target and/or different on-target activities [4]. However, the application of computational tools for rational drug discovery and repurposing has significantly increased over the last years, along with technological improvements and the availability of public databases [5,6,7], further advances in medicinal chemistry approaches, and the development of dedicated hardware and software tools [8]. At present, such approaches allow a more in-depth exploitation of chemical, biological and structural data useful for a more efficient drug repositioning campaign. A vast number of computational methodologies are now available to advance drug repurposing projects on rational grounds. For example, the identification of novel targets and therapeutic indications for already-known molecules can rely on artificial intelligence (AI) approaches, molecular modeling techniques, and chemoinformatics tools [8,9,10,11,12]. Of note, their use in integrated workflows and their ability to complement each other enable overcoming limitations inherent to each approach [10].

On these premises, this Special Issue entitled “Trends and Applications in Computationally Driven Drug Repurposing” is focused on current approaches and methods available for computational drug repurposing, their integration into innovative and tailored in silico strategies and workflows, and their exploitation for the efficient analysis of information reported in databases. In addition, this Special Issue is tailored to in silico approaches focusing on target-based and phenotypic-based screenings for drug repurposing, including the analysis of metabolic networks. This Special Issue included a total of five contributions, four of them being original articles and one being a review, collectively providing useful hints and future perspectives regarding computational drug repurposing. In particular, Flori et al. [13] reported the development and application of an in silico approach for the repurposing of drugs approved by FDA as potential interfering agents in the formation of irisin dimers, which are involved in lipodystrophy (LD) syndromes when present at aberrant levels in cells. To this end, the authors performed docking-based screenings of irisin monomers, followed by molecular dynamics (MD) simulations, and binding free energy- and HADDOCK-based [14] calculations. The performed analyses allowed the identification of five candidates (iohexol, paromomycin, setmelanotide, theophylline and zoledronate) as putative irisin disruptors. In this article, the authors highlighted the utility of selected candidates in both drug repurposing and repositioning contexts, the latter approach allowing their full exploitation for discovering new active pharmaceutical ingredients. In addition, this study highlighted how different in silico approaches can complement each other in tailored workflows to improve the prediction performance of virtual screening campaigns.

Similar considerations arise from the study of Bernal et al. [15], in which different types of ligand-based similarity estimations were performed between molecules with reported antiproliferative activity against prostate cancer (PC) cells and DrugBank compounds [16]. In this study, the performed analyses were also integrated with target-related activity data reported for PC, allowing the authors to identify 48 DrugBank compounds as potential repurposing or repositioning candidates, 5 of which have already been studied in clinical trials for prostate cancer. Moreover, 10 candidates with potent activity on PC cells and proven safety profile, as assessed through a phase I clinical study, were also identified; these compounds were therefore selected as suitable candidates for fast repurposing against PC. Notably, these drugs are effective through the modulation of targets and pathways that are highly relevant in prostate cancer.

In recent times, there has been increased interest around proteolysis-targeting chimeras (PROTACs) as novel agents for the treatment of a variety of human diseases [17,18,19]; such compounds enable the exploitation of the ubiquitin–proteasome system (UPS) for selectively degrading disease-related proteins. In this context, significant opportunities can be derived by means of the repositioning of already reported PROTAC therapeutics towards different players in the ubiquitination process. Palomba et al. [20] provided a very interesting example in this respect, searching for novel E3 ligases through a scaffold-repurposing strategy. In particular, they first applied their ELIOT (E3 LIgase pocketOme navigaTor) platform [21] in search of cross-relationships between the binding site of E3 ligases tripartite motif containing 24 (TRIM24) and tripartite motif containing 33 (TRIM33), identifying scaffolds and structural motifs present in known TRIM24 ligands, and potentially exploitable for repositioning strategies towards TRIM33. Afterwards, the authors performed docking analyses in order to identify a series of ligands to be synthesized and tested against TRIM33; notably, for the first time, X-ray complexes of TRIM33 with three of the obtained compounds were also obtained. Altogether, these results suggest that the design of novel and challenging PROTAC ligands can be sensibly sped up by means of scaffold-repurposing strategies.

Lam and coworkers [22] reported the application of a series of computational approaches in the search for compounds inhibiting E8, which is a protein necessary for the monkeypox virus (MPVX) to attach to the glycosaminoglycan (GAG) adhesion molecules present on the human cell’s surface [22,23]. While no crystal structure of the MPXV E8 protein was available at the time of the study, its three-dimensional model has been derived by Alphafold server [24], an AI-based software which has recently revolutionized the homology modelling of proteins with yet unsolved 3D structure. Docking calculations followed by MD simulations and MM-GBSA analyses have been performed on the Alphafold-derived 3D model, allowing the identification of Diosmin and Flavin adenine dinucleotide (FAD) as putative repurposing candidates against the E8 viral protein; the inhibition of this target could decrease the transmission rate of the monkeypox virus, which became a worldwide public health emergency in 2022 according to the World Health Organization.

The review article by Van Tran et al. [25] focused on trends and advances in computationally based drug distribution predictions, while also discussing the key influencing factors, recent databases, and AI-powered models used for this purpose. The authors described recent tools for the prediction of blood–brain barrier permeability, plasma protein binding, fractions unbound in plasma and the volume of distribution, while also discussing how improvements in data diversification, data-cleaning and preprocessing, and the development of more sophisticated and robust AI algorithms could help to improve the prediction performance of the pharmacokinetic profiles of drugs. The early prediction of the absorption, distribution, metabolism, and excretion (ADME) profiles of compounds is of primary interest in the de novo design of drug candidates, especially considering that many compounds that reach clinical trials fail due to unexpected pharmacokinetic issues [26]. These aspects are also of relevance in drug repurposing and repositioning contexts, considering that the safety requirements for drug approval may differ depending on the therapeutic area. Similar considerations can arise with regard to the ADME profile of the compounds, whose efficacy as repurposing candidates against different pathologies might also depend, for example, on their distribution in specific tissues.

Drug repurposing has attracted increasing interests in both the pharmaceutical industry and academia. Compared to other technologies or discovery strategies, it allows practitioners to reduce costs and failures. Indeed, a repositioning campaign—from the identification of the compound to its introduction on the market—requires on average from 3 to 12 years, and an investment of USD 300 million, compared to 10–17 years and USD 2–3 billion in the case of the launch of an entirely new chemical entity [4]. In the era of big data [27,28,29], data science, and artificial intelligence [30], computational technologies have progressed to a higher level of predictive ability, thus representing an increasingly attractive avenue to further reduce costs and risks of drug repurposing approaches [9]. Overall, the collection of articles reported in this Special Issue provides an interesting view on how different computational approaches, including structure-based methods, chemoinformatics and AI, can be efficiently integrated to advance and speed-up various tasks involved in the rational drug repurposing process. On these grounds, computationally driven drug repurposing will play a central role in the future discovery of therapeutics for established and emerging targets and diseases.
